# Low-density lipoprotein cholesterol goal attainment rates in high-risk patients with cardiovascular diseases and diabetes mellitus in Korea: a retrospective cohort study

**DOI:** 10.1186/s12944-019-1158-5

**Published:** 2020-01-11

**Authors:** Ye Seul Yang, Bo Ram Yang, Mi-Sook Kim, Yunji Hwang, Sung Hee Choi

**Affiliations:** 1 Department of Internal Medicine, Seoul National University College of Medicine, 101 Daehak-ro, Jongno-gu, Seoul, Republic of Korea; 2 Medical Research Collaborating Center, Seoul National University Hospital, 101 Daehak-ro, Jongno-gu, Seoul, Republic of Korea; 3 Department of Preventive Medicine, Seoul National University College of Medicine, 101 Daehak-ro, Jongno-gu, Seoul, Republic of Korea; 4 Amgen Korea, 19 Eulji-ro 5-gil, Jung-gu, Seoul, Republic of Korea; 5 Department of Internal Medicine, Division of Endocrinology & Metabolism, Seoul National University Bundang Hospital, 82, Gumi-ro 173Beon-gil, Bundang-gu, Seongnam-si, Gyeonggi-do Republic of Korea

**Keywords:** Dyslipidaemia, Low density lipoprotein cholesterol, Stroke, Acute coronary syndrome, Cardiovascular disease, Diabetes mellitus, Statin

## Abstract

**Background:**

Real-world evidence of low-density lipoprotein cholesterol (LDL-C) goal attainment rates for Asian patients is deficient. The objective of this study was to assess the status of dyslipidemia management, especially in high-risk patients with cardiovascular disease (CVD) including stroke and acute coronary syndrome (ACS).

**Methods:**

This was a retrospective cohort study of 514,866 subjects from the National Health Insurance Service-National Health Screening Cohort database in Korea. Participants were followed up from 2002 to 2015. Subjects with a high-risk of CVD prior to LDL-C measurement and subjects who were newly-diagnosed for high-risk of CVD following LDL-C measurement were defined as known high-risk patients (*n* = 224,837) and newly defined high-risk patients (*n* = 127,559), respectively. Data were analyzed by disease status: stroke, ACS, coronary heart disease (CHD), peripheral artery disease (PAD), diabetes mellitus (DM) and atherosclerotic artery disease (AAD).

**Results:**

Overall, less than 50% of patients in each disease category achieved LDL-C goals (LDL-C < 70 mg/dL in patients with stroke, ACS, CHD and PAD; and LDL-C < 100 mg/dL in patients with DM and AAD). Statin use was observed in relatively low proportions of subjects (21.5% [known high-risk], 34.4% [newly defined high-risk]). LDL-C goal attainment from 2009 to 2015 steadily increased but the goal-achiever proportion of newly defined high-risk patients with ACS remained reasonably constant (38.7% in 2009; 38.1% in 2015).

**Conclusions:**

LDL-C goal attainment rates in high-risk patients with CVD and DM in Korea demonstrate unmet medical needs. Proactive management is necessary to bridge the gap between the recommendations of clinical guidelines and actual clinical practice.

## Introduction

Cardiovascular disease (CVD) is the leading cause of death globally, with 17.9 million estimated deaths from CVD in 2016, representing 31% of all global deaths. Myocardial infarction and stroke account for 85% of CVD deaths [1]. Dyslipidemia is a major risk factor for coronary heart disease (CHD) and stroke [2–5], and includes elevated total cholesterol, triglycerides, or low-density lipoprotein cholesterol (LDL-C) levels, or low high-density lipoprotein cholesterol (HDL-C) levels. The global disease burden of CVD increased by 12.5% [6], and this trend is attributed by Asians with fast growing of aged population [7]. However, evidence is limited for dyslipidemia management for high-risk CVD patients among Asians. Recent data from the Korean National Health and Nutrition Examination Survey (KNHANES) reported that, in 2016, 19.9% of adults aged ≥30 years had hypercholesteremia and 40.5% had dyslipidemia [8]. The prevalence of dyslipidemia in Korea has increased in an age-dependent manner and is more evident in women aged ≥50 years [8–11]. The level of disease awareness was as low as 32.1% in men and 32.6% in women (aged 30–49 years) [11]. In one recent study in Korea, the prevalence of dyslipidemia was higher than that of hypertension and diabetes mellitus (DM), but dyslipidemia awareness and treatment rates were still lower [12].

LDL-C remains the primary target of cholesterol-lowering therapy for the primary and secondary prevention of atherosclerotic cardiovascular disease (ASCVD) events including CHD, stroke, and peripheral artery disease (PAD). The cardiovascular risk level of individuals determines LDL-C treatment goals [2–4, 13–15]. Some differences in cholesterol-lowering guidelines have been described and these may, at least in part, be attributable to whether the guidelines are solely evidence-based or based on a combination of evidence and expert opinion [16, 17]. On the other hand, management of dyslipidemia was revolutionized since statins were discovered. Statins are known to substantially reduce LDL-C levels and CVD mortality [18, 19]. Previous studies have shown that further reductions in LDL-C levels by more intensive statin therapy according to the risk of CVD have further benefits [20, 21].

American College of Cardiology/American Heart Association (ACC/AHA) guidelines [3] emphasized > 50% LDL-C reductions from baseline in high-risk patients. European Society of Cardiology and European Atherosclerosis Society (ESC/EAS) guidelines in 2016 focused on < 70 mg/dL LDL-C reductions in high-risk patients [15]. Recently revised 2018 ACC/AHA guidelines emphasize using an LDL-C threshold of 70 mg/dL for considering the addition of non-statins to statin therapy in very high-risk patients, including a history of multiple major ASCVD events or one major ASCVD event and multiple high-risk conditions [22]. This means that, in addition to percent LDL-C reductions from baseline, target LDL-C levels are also critical values as LDL-C treatment goals for dyslipidemia management. The latest Korean national guidelines were formulated from the Committee of Clinical Practice of the Korean Society of Lipid and Atherosclerosis for the Management of Dyslipidemia in 2018 [23]. It was generally based on the third report of the National Cholesterol Education Program-Adult Treatment Panel (NECP-ATP) [2] for the risk stratification and LDL-C treatment goal. However, > 50% LDL-C reduction was recommended according to ACC/AHA guidelines in case of ACS patients in addition to the LDL-C target < 70 mg/dL and intensity of statin also was recommend in accordance with the ACC/AHA guidelines [3]. The target of the recent American Association of Clinical Endocrinologists guideline [4] for lowering LDL-C to less than 55 for extreme high risk was not included in the recommendations yet as consensus from domestic experts will be required.

Evidence of LDL-C goal attainment rates for Korean patients compared with other recently updated guidelines is currently lacking, particularly in high-risk patients. Therefore, it is needed to evaluate the status of dyslipidemia management in Korea in general, as well as specifically addressing the status of high-risk patients with stroke, acute coronary syndrome (ACS), CHD, PAD, DM and atherosclerotic artery disease (AAD). This study used absolute values for LDL-C level < 70 mg/dL in patients with very-high risk disease (stroke, ACS, CHD and PAD) and < 100 mg/dL LDL-C in high-risk patients (DM and AAD); and > 50% reduction using repeated measured LDL-C levels, as LDL-C treatment goals. Also, this study aimed to describe the time trends of LDL-C goal attainment rate in recent years using extensive national data. To identify the most appropriate populations to target with preventative therapies, subgroup analyses were conducted.

## Methods

### Study design

This retrospective cohort study used data from the Korean National Health Insurance Service-National Health Screening Cohort (NHIS-HEALS), details of which have been described elsewhere [24]. The NHIS has provided a general national health screening program since 1995, and a health screening program for transitional ages, aimed at individuals aged 40 and 66 years, since 2007. The general health screening program is applied at least once every 2 years for the entire population of Korean adults aged ≥40 years; the participation rate was 74.8% in 2014. NHIS-HEALS incorporates information from these health screening programs [25].

The NHIS-HEALS database comprised 514,866 subjects (aged 40–79 years, 54.2% males) at baseline (2002–2003) who were randomly selected by simple random sampling using SAS version 9.4 (SAS Institute Inc., Cary, NC, USA) and represented 10% of all national health screening participants (*N* = 5,148,695) in 2002 and 2003. Participants were followed up from 2002 to 2015 and data constructed in 2015. Variables included social and economic qualifications, medical check-up results, healthcare usage and survival status linked to national death certificates [24]. The healthcare usage database included information on records of inpatient and outpatient usage (diagnosis, procedures, and prescriptions). Diagnoses were coded according to the International Classification of Disease, Tenth Revision (ICD-10) [26].

### Risk stratification

Korean national guidelines [23] based on the risk stratification of NCEP-ATP III, categorized risk groups to very high-risk, high-risk, moderate-risk and low-risk and recommended LDL-C treatment goals dependent on risk assessment: very high-risk < 70 mg/dL, high-risk < 100 mg/dL, moderate-risk < 130 mg/dL, and low-risk < 160 mg/dL. Very high-risk consisted of ACS, stroke and TIA, and PAD; high-risk consisted of carotid artery disease, abdominal aortic aneurysm, and DM. According to guidelines, PAD and other AAD (including carotid artery disease and abdominal aortic aneurysm) were separated to adjust different LDL-C target goal. ICD-10 codes and related procedures for risk stratification were listed in Table 1.

**Table 1:** Definition of high-risk disease by ICD-10 codes and procedure code

Disease	Diagnosis or procedure code
Stroke	Diagnosis: I63^a^, I64^a^, I69.3^b^, G45, G46
ACS	Diagnosis: I21^a^, I22^a^, I23
	Procedure:
	Coronary artery bypass graft: OA640–2, OA647–9, O1640–2, O1647–9
	Percutaneous coronary intervention: M6561–7, M6571–2
	Percutaneous transluminal coronary angioplasty: M6551–4
CHD	Diagnosis: I20.0, I20.9, I24.0, I25.1, I25.2^b^, I25.5, I25.6
PAD	Diagnosis: I70.2, I73.1, I73.8, I73.9
DM	Diagnosis: E10, E11, E12, E13, E14
AAD	Diagnosis: I65.2, I71.3, I71.4, I71.5, I71.6

*AAD* atherosclerotic artery disease, *ACS* acute coronary syndrome, *CHD* coronary heart disease, *DM* diabetes mellitus, *ICD-10* International Classification of Diseases (10th revision), *PAD* peripheral artery disease

^a^Included only in the case of hospitalization; ^b^Included for Known high risk

### Eligibility criteria

**Fig. 1:**
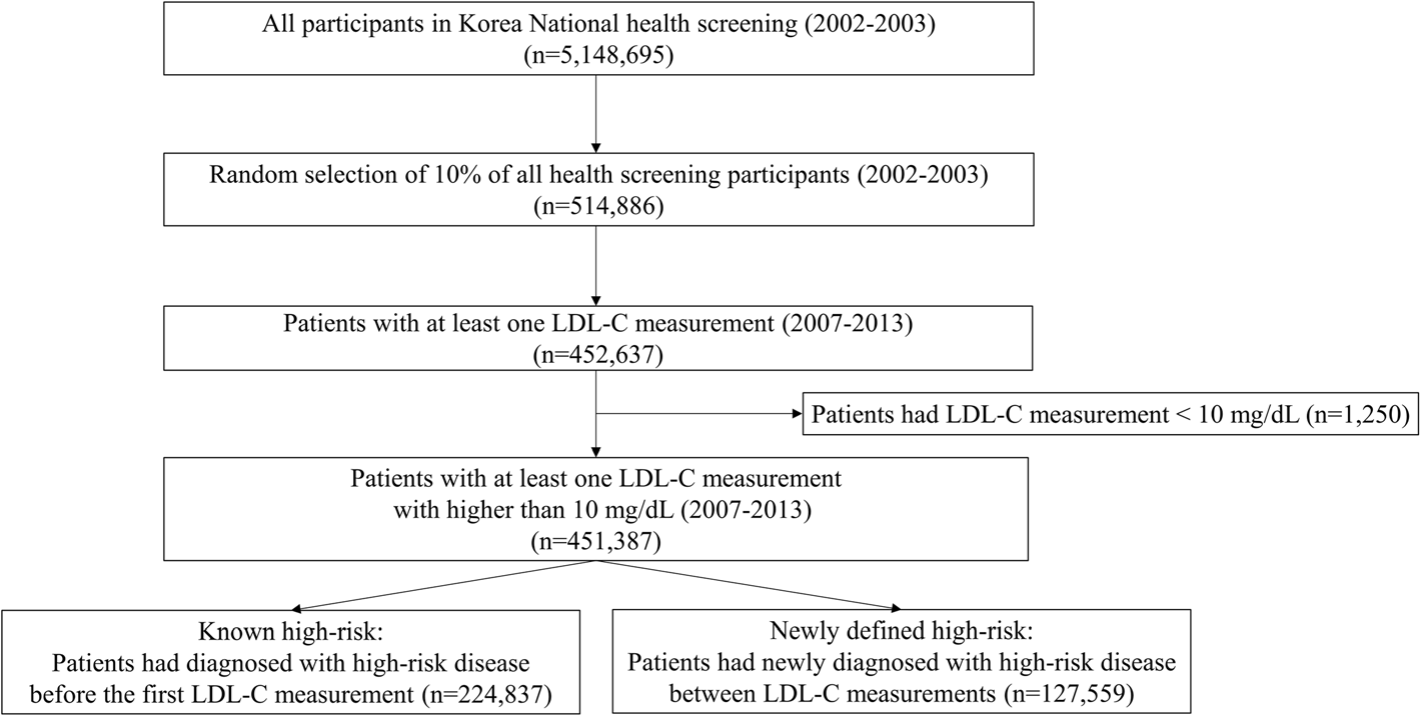
Flow chart of the selection of high-risk category subjects

From the NHIS-HEALS database, patients with LDL-C measurements during 2007–2013 were included. Although data on participants’ total cholesterol levels are available from 2002, data for triglyceride, HDL-C and LDL-C levels are available for the health screening program for transitional ages from 2007, and for general national health screening programs from 2009. Patients with LDL-C measurement < 10 mg/dL during 2007–2013 were excluded. Subjects with a high-risk of CVD including stroke, ACS, CHD, PAD, DM and AAD were identified using ICD-10 codes and related procedures (Table 1) and classified into two groups: 1) known high-risk patients, 2) newly defined high-risk patients (Fig. 1).

Subjects previously identified as having a high-risk for CVD, prior to measurement of LDL-C levels, were categorized as “known high-risk patients.” The index date is the first LDL-C testing date during the study period. Definition of high-risk status was required to be made in the previous year including the index date. Subjects categorized as “newly defined high-risk” were identified according to the following criteria: 1) patients with more than two LDL-C measurements, and 2) patients who were newly diagnosed or underwent procedures for high-risk CVD between two LDL-C measurements. As LDL-C levels were available from 2007, patients with new cases of each disease could be defined as having at least 5 years of disease-free periods. For subjects with newly defined high-risk disease, the earliest date of visit regarding high-risk disease was defined as the index date. Here is an example of the group definition. If a subject had five LDL-C measurements during the follow up period and there was a first diagnosis of DM prior to the first LDL-C measurement, he or she was defined as a known high-risk patient for DM; on the other hand, there was a first ACS diagnosis between the third and fourth LDL-C measurements, he or she was defined as a newly diagnosed high-risk patient for ACS.

### Outcome variables: LDL-C goal attainment

Target LDL-C levels were defined by the 2018 Korean guidelines [23]. For patients with stroke, ACS, CHD or PAD, defined as the very high-risk group, the target level was < 70 mg/dL. For patients with DM or AAD, defined as high-risk group, the target level was < 100 mg/dL. When patients with DM or AAD had other concurrent high-risk diseases—stroke, ACS, CHD or PAD—these patients were stratified into the subgroups “DM with high-risk of CVD” and “AAD with high-risk of CVD” for outcome analysis, because their target level was < 70 mg/dL as very high-risk group. To determine LDL-C target achievement, LDL-C levels at the index date and LDL-C levels after the index date were used for known high-risk patients and newly defined high-risk patients, respectively.

LDL-C goal attainment by reduction rates were defined by 2013 ACC/AHA guidelines: > 50% reduction in baseline LDL-C for high-intensity statin and 30 to < 50% for moderate-intensity statin; guidelines recommend high-intensity statin therapy in patients with ASCVD or those with DM aged > 45 years [3]. In the present study, the two LDL-C levels just before and after the index date of newly defined high-risk patients were used to calculate changes in LDL-C levels. On average, the interval for the two LDL-C tests was approximately 1 year, due to government policy for providing the health screening program.

### Assessment of statin use

Statins included atorvastatin, fluvastatin, lovastatin, pitavastatin, pravastatin, rosuvastatin and simvastatin. Statin therapy intensity was classified as high-intensity (atorvastatin 40–80 mg, rosuvastatin 20 mg), moderate-intensity (atorvastatin 10–20 mg, fluvastatin 40–80 mg, lovastatin 40 mg, pitavastatin 2–4 mg, pravastatin 40 mg, rosuvastatin 5–10 mg, simvastatin 20–40 mg), and low-intensity (fluvastatin 20–40 mg, lovastatin 20 mg, pitavastatin 1 mg, pravastatin 5–20 mg, simvastatin 5–10 mg), according to generic name and dose.

Statin exposure was assessed on or within 30 days before the index date using the prescription date and duration in known high-risk patients. For newly defined high-risk patients, whether exposure to statin took place from 30 days before to 90 days after the index date, using prescription dates and duration was assessed. Subjects were classified by their statin use history, as existing users if receiving statins at the time of the index date or within 6 months before the index date, or new users if there was no record of statin use for 6 months prior to the index date.

### Data analysis

General characteristics including age, sex, body mass index (BMI), waist circumference, smoking, presence of diabetes, presence of hypertension, systolic and diastolic blood pressure measurements, fasting blood glucose levels, total cholesterol, triglyceride, HDL-C and LDL-C, are presented as mean and standard deviation (SD) for continuous variables and as frequency and proportion for categorical variables.

The proportion of patients attaining target LDL-C levels was calculated by dividing the number of patients with LDL-C level less than target level by the total number of patients. The proportion of patients with > 50% reduction of LDL-C levels was calculated by dividing the number of patients with > 50% reduction of LDL-C from the previous LDL-C level by the total number of patients. All LDL-C measurements from the index date were included to identify annual trend. Data were analyzed by disease status: stroke, ACS, CHD, PAD, DM and AAD. Data were analyzed using SAS Enterprise Guide 7.1 (SAS Institute, Cary, North Carolina, USA).

## Results

### Patients’ characteristics

The NHIS-HEALS database (*n* = 514,866) included high-risk patients either before LDL-C measurement (known high-risk; *n* = 224,837) or following LDL-C measurement (newly defined high-risk; *n* = 127,559) (Fig. 1). Baseline characteristics of subjects stratified by cardiovascular risk category (known or newly defined high-risk), and by disease, are shown in Tables 2 and 3. DM was the most common disease in the known high-risk group (*n* = 153,050), followed by PAD (*n* = 89,807), and CHD (*n* = 65,868). In patients with newly defined high-risk for CVD, PAD (*n* = 55,767) was the most common disease followed by DM (*n* = 52,416), and CHD (*n* = 29,434) (Tables 2 and 3). Mean age was similar across patient groups (62–66 years; known high-risk and newly defined high-risk). For ACS (67.89%), CHD (52.82%), DM (52.31%) and AAD (62.87%), more than half of all patients were men; for stroke (49.53%) and PAD (47.66%), less than half of the patients were men.

**Table 2:** Baseline characteristics in subjects stratified by cardiovascular risk category and disease: known high-risk patients

	Stroke	ACS	CHD	PAD	DM	AAD
	(n = 39,317)	(n = 5309)	(n = 65,868)	(n = 89,807)	(n = 153,050)	(*n* = 2200)
Mean age, years (±SD)	65.39 ± 9.43	65.09 ± 9.39	63.41 ± 9.21	63.79 ± 9.39	62.63 ± 9.11	66.45 ± 9.02
Male, *n* (%)	18,575 (47.24)	3442 (64.83)	34,431 (52.27)	38,262 (42.6)	80,104 (52.34)	1380 (62.73)
Mean BMI, kg/m^2^ (±SD)	24.28 ± 3.16	24.35 ± 3.16	24.51 ± 3.08	24.33 ± 3.15	24.39 ± 3.11	24.05 ± 3.06
Mean waist circumference, cm (±SD)	83.5 ± 8.52	84.69 ± 8.37	83.94 ± 8.42	83.06 ± 8.5	83.69 ± 8.43	84.19 ± 8.47
Smoking, *n* (%)						
non-smoker	27,731 (71.56)	3029 (58.07)	43,183 (66.65)	64,638 (72.92)	99,636 (66.21)	1291 (59.69)
former smoker	6549 (16.90)	1370 (26.27)	12,980 (20.03)	12,987 (14.65)	27,958 (18.58)	533 (24.64)
current smoker	4474 (11.54)	817 (15.66)	8625 (13.31)	11,012 (12.42)	22,887 (15.21)	339 (15.67)
DM, *n* (%)	23,175 (58.94)	3806 (71.69)	38,946 (59.13)	46,651 (51.95)	153,050 (100.0)	1477 (67.14)
Hypertension, *n* (%)	30,727 (78.15)	4759 (89.64)	52,413 (79.57)	61,991 (69.03)	102,151 (66.74)	1918 (87.18)
Mean systolic BP, mmHg (±SD)	128.08 ± 15.79	126.98 ± 16.2	127.34 ± 15.54	127.71 ± 15.71	127.37 ± 15.5	128.45 ± 16.43
Mean diastolic BP, mmHg (±SD)	78.06 ± 10.06	77.11 ± 10.24	77.8 ± 9.99	78.1 ± 9.99	77.9 ± 9.91	77.51 ± 10.23
Mean total cholesterol, mg/dL (±SD)	194.21 ± 40.18	177.03 ± 40.72	191.38 ± 39.68	198.2 ± 39.88	195.06 ± 39.69	183.39 ± 41.46
Mean triglycerides, mg/dL (±SD)	141.79 ± 83.48	139.2 ± 79.8	140.41 ± 83.85	141.98 ± 84.27	144.76 ± 88.7	134.63 ± 75.27
Mean HDL-C level, mg/dL (±SD)	53.6 ± 31.11	52.22 ± 36.79	53.45 ± 28.84	54.2 ± 27.02	53.47 ± 27.19	51.48 ± 21.36
Mean LDL-C level, mg/dL (±SD)	114.02 ± 38.97	99.71 ± 39.85	111.47 ± 39.52	116.79 ± 38.37	113.99 ± 38.62	106.1 ± 41.52
Mean fasting plasma glucose, mg/dL (±SD)	104.49 ± 29.4	108.7 ± 35.96	105.12 ± 29.52	104.62 ± 29.76	111.58 ± 35.65	106.7 ± 31.86

* Variables from the health screening program were included missing data. Missing rates (%) are 0.08 for BMI, 0.10 for waist circumference, 1.55 for Smoking, 0.04 for systolic BP, 0.04 for diastolic BP, 0.001 for total cholesterol, 0.08 for triglycerides, 0.004 for HDL-C level, 0.000 for LDL-C, and 0.002 for fasting plasma glucose

*AAD* atherosclerotic artery disease, *ACS* acute coronary syndrome, *CHD* coronary heart disease, *DM* diabetes mellitus, *PAD* peripheral artery disease

**Table 3:** Baseline characteristics in subjects stratified by cardiovascular risk category and disease: newly defined high-risk patients

	Stroke	ACS	CHD	PAD	DM	AAD
	(*n* = 17,410)	(*n* = 2479)	(*n* = 29,434)	(*n* = 55,767)	(*n* = 52,416)	(*n* = 4624)
Mean age, years (±SD)	65.22 ± 8.76	65.71 ± 8.8	63.61 ± 8.51	63.21 ± 8.41	61.92 ± 8.3	65.74 ± 8.18
Male, *n* (%)	8623 (49.53)	1683 (67.89)	15,547 (52.82)	26,576 (47.66)	27,417 (52.31)	2907 (62.87)
Mean BMI, kg/m^2^ (±SD)	24.2 ± 2.96	24.47 ± 3.01	24.42 ± 3	24.25 ± 2.95	24.41 ± 3	24.21 ± 2.81
Mean waist circumference, cm (±SD)	82.99 ± 8.31	84.94 ± 8	83.44 ± 8.28	82.73 ± 8.29	83.09 ± 8.33	83.72 ± 8.07
Smoking, *n* (%)						
non-smoker	11,835 (68.66)	1294 (52.84)	19,085 (65.55)	38,415 (69.63)	33,675 (64.96)	2636 (57.57)
former smoker	3015 (17.49)	536 (21.89)	5679 (19.5)	9508 (17.23)	10,006 (19.3)	1133 (24.74)
current smoker	2387 (13.85)	619 (25.28)	4353 (14.95)	7249 (13.14)	8162 (15.74)	810 (17.69)
DM, *n* (%)	8393 (48.21)	1274 (51.39)	13,860 (47.09)	24,589 (44.09)	0 (0.00)	2650 (57.31)
Hypertension, *n* (%)	10,910 (62.67)	1708 (68.90)	18,210 (61.87)	32,098 (57.59)	26,890 (51.30)	3300 (71.37)
Mean systolic BP, mmHg (±SD)	127.83 ± 15.67	129.22 ± 15.25	127.53 ± 15.42	126.89 ± 15.48	127.13 ± 15.46	128.4 ± 15.15
Mean diastolic BP, mmHg (±SD)	78.18 ± 10.04	78.41 ± 9.85	78.26 ± 10.03	77.86 ± 9.96	78.48 ± 10.06	77.78 ± 9.84
Mean total cholesterol, mg/dL (±SD)	199.09 ± 39	202.64 ± 43.09	199.54 ± 38.93	199.22 ± 38.59	203.88 ± 39.46	195.52 ± 40.2
Mean triglycerides, mg/dL (±SD)	140.53 ± 82.41	153.91 ± 88.65	141.5 ± 84.77	138.95 ± 83.78	146.67 ± 91.49	139.7 ± 80.57
Mean HDL-C level, mg/dL (±SD)	53.49 ± 23.68	50.51 ± 18.84	53.69 ± 24.23	54.42 ± 24.76	54.15 ± 22.38	52.06 ± 16.44
Mean LDL-C level, mg/dL (±SD)	118.37 ± 37.42	121.51 ± 39.64	118.52 ± 37.17	117.99 ± 37	121.2 ± 37.28	115.85 ± 37.4
Mean fasting plasma glucose, mg/dL (±SD)	103.58 ± 28.42	107.9 ± 33.18	103.76 ± 27.51	103.15 ± 27.36	106.94 ± 30.32	106.46 ± 29.8

* Variables from the health screening program were included missing data. Missing rates (%) for variables are 0.01 for BMI, 0.03 for waist circumference, 0.72 for Smoking, 0.02 for systolic BP, 0.02 for diastolic BP, 0.000 for total cholesterol, 0.09 for triglycerides, 0.002 for HDL-C level, 0.000 for LDL-C, and 0.001 for fasting plasma glucose

*AAD* atherosclerotic artery disease, *ACS* acute coronary syndrome, *CHD* coronary heart disease, *DM* diabetes mellitus, *PAD* peripheral artery disease

### Statin use

Relatively low proportions of subjects were under lipid-lowering therapy with statin (21.5% [known high-risk], 34.4% [newly defined high-risk]). Among statin users, most patients in both the known and newly defined high-risk groups received moderate-intensity statin therapy (89.3–92.5%). High-intensity statin therapy was least commonly used in known high-risk patients, but more frequent in newly defined high-risk patients: 2.8 and 12.5% (stroke), 6.1 and 32.0% (ACS), 2.7 and 8.3% (CHD), 1.8 and 3.0% (PAD), 2.0 and 4.4% (DM), and 3.3 and 10.4% (AAD), respectively.

**Fig. 2:**
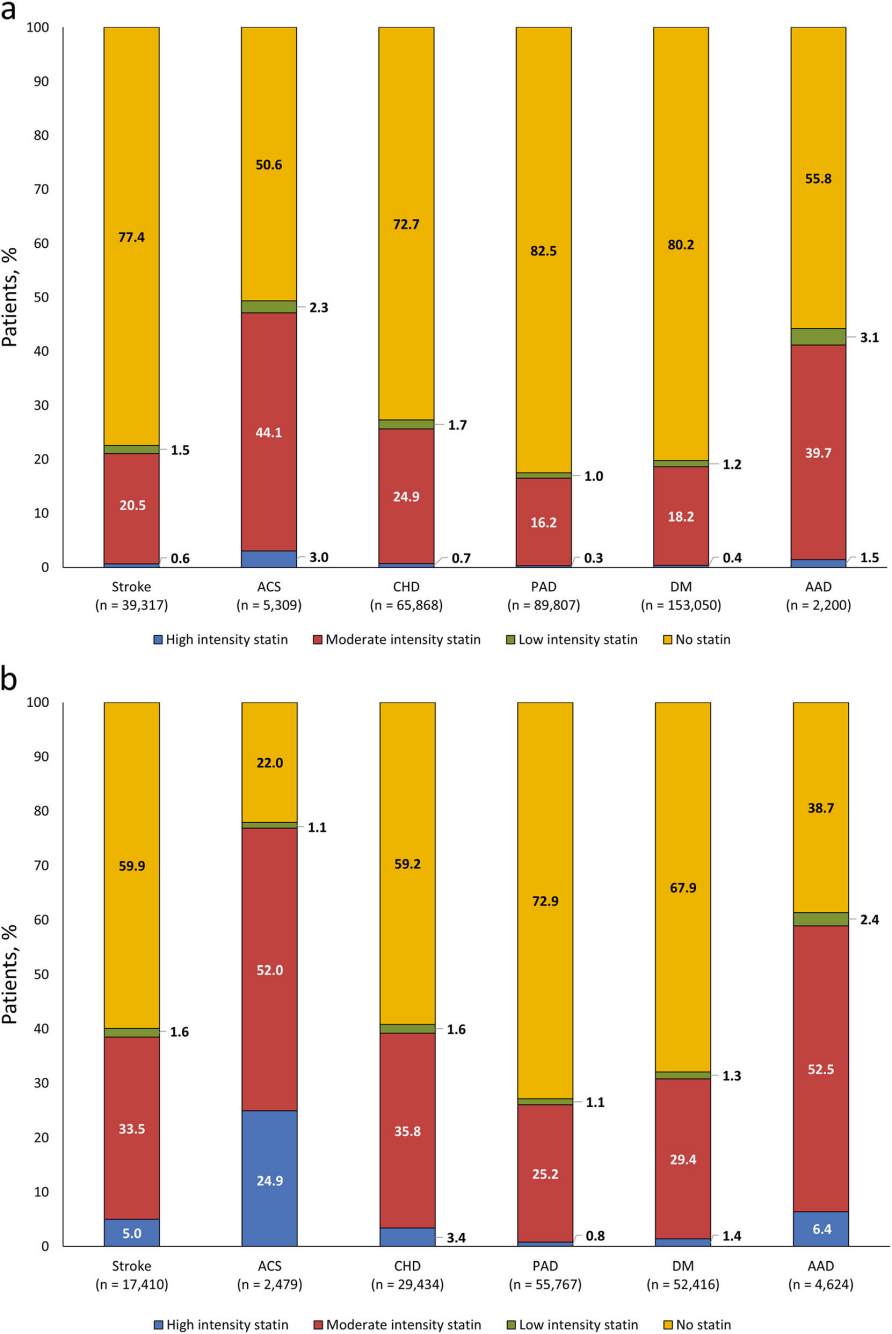
Statin use in cardiovascular high-risk groups: **a.** in known high-risk patients and **b.** in newly defined high-risk patients. AAD, atherosclerotic artery disease; ACS, acute coronary syndrome; CHD, coronary heart disease; DM, diabetes mellitus; PAD, peripheral artery disease

Overall, 22.6 and 40.1% of known high-risk and newly defined high-risk stroke patients, respectively, received statin therapy (Fig. 2). For stroke patients defined as the known high-risk group (*n* = 39,317), 1.5, 20.5 and 0.6% received low-, moderate- or high-intensity statin therapy, respectively. For stroke patients defined as the newly defined high-risk (*n* = 17,410), corresponding values were 1.6, 33.5 and 5.0%. Overall, 49.4% of ACS patients with known high-risk received statin therapy versus 78.0% of patients with newly defined high-risk. In ACS patients with known high-risk (*n* = 5309), 2.3, 44.1 and 3.0% received low-, moderate- or high-intensity statin therapy, respectively; corresponding values were 1.1, 52.0 and 24.9%, in ACS patients with newly defined high-risk (*n* = 2479). This difference was largely due to a higher proportion of newly defined high-risk patients receiving high-intensity statin therapy compared with known high-risk patients (24.9% vs. 3.0%) (Fig. 2).

**Fig. 3:**
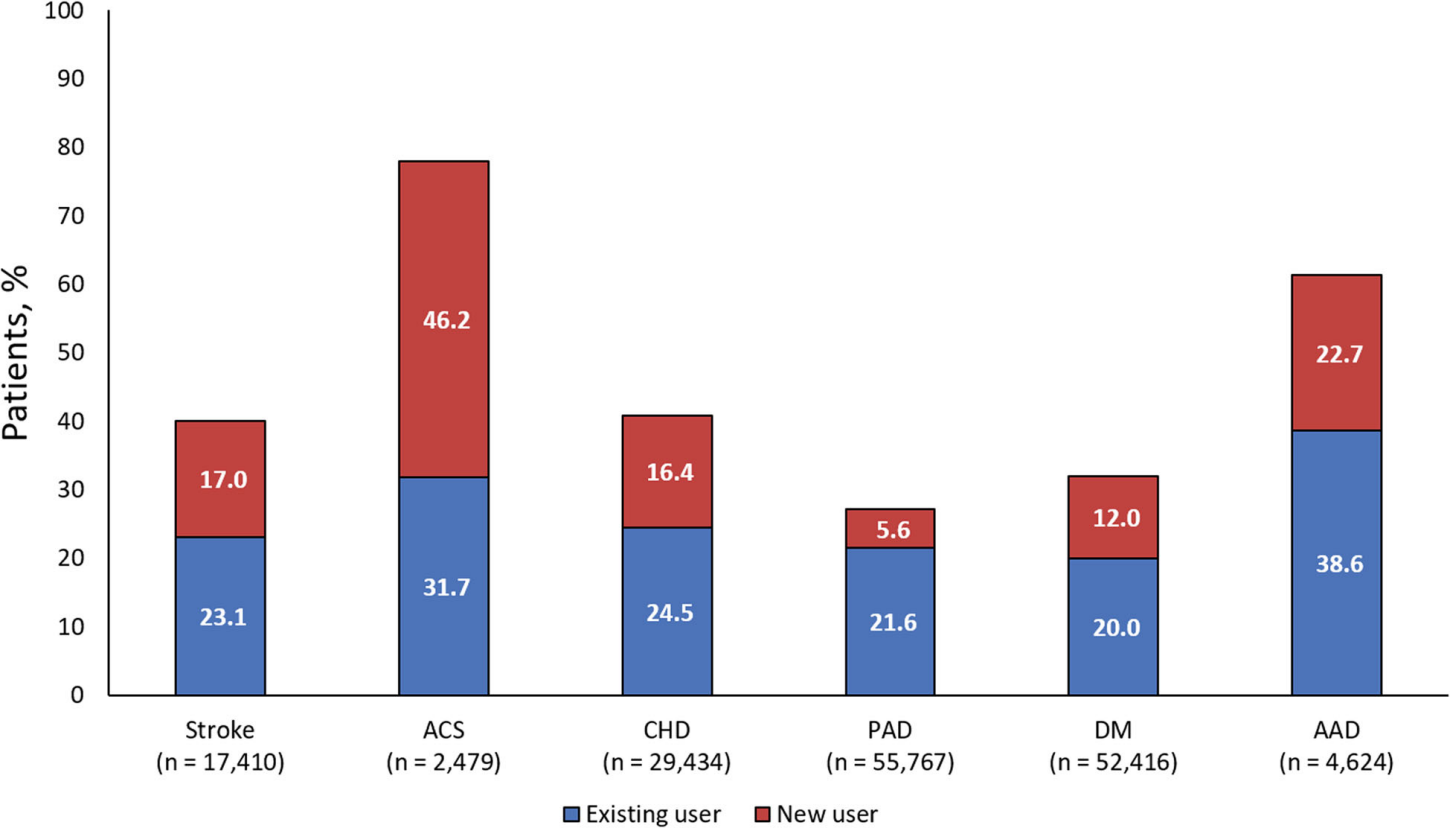
Patients previously receiving statins (existing user) or newly prescribed statins (new user)

Statins were also used more frequently in newly defined high-risk patients compared with known high-risk patients in CHD (40.8% vs. 27.3%), PAD (27.1% vs. 17.5%), DM (32.1% vs. 19.8%) and AAD (61.3% vs. 44.2%) (Fig. 2). In newly defined high-risk stroke patients (*n* = 17,410), 23.1% were existing statin users and 17.0% were new statin users. In newly defined high-risk ACS patients (*n* = 2479), 31.7% were existing users, and 46.2% were new users (Fig. 3).

### LDL-C goal attainment rates

**Fig. 4:**
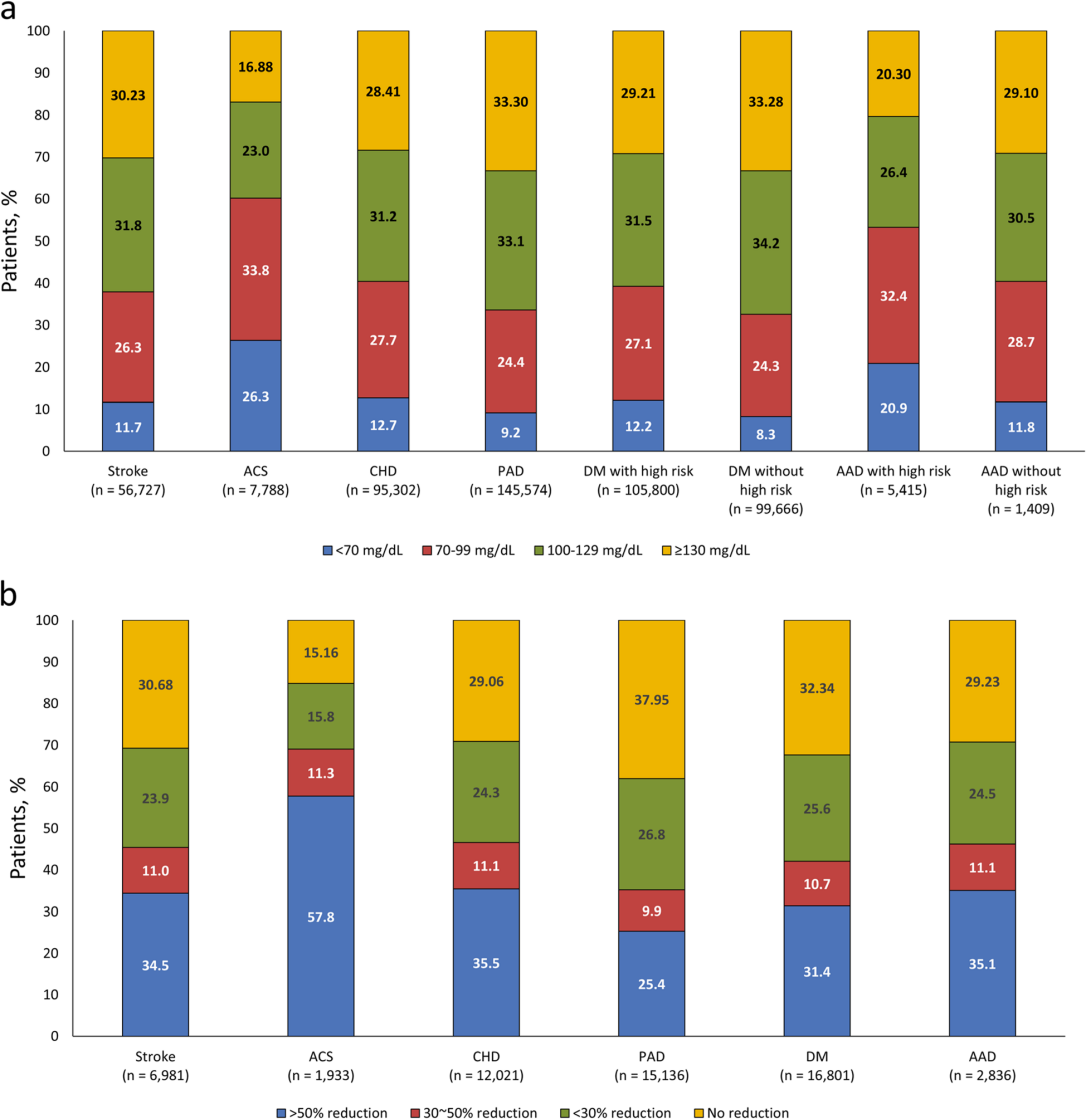
LDL-C goal attainment rates in **a.** all high-risk (known + newly defined high-risk patients) by target LDL-C level and **b.** in newly defined high-risk patients stratified by disease by reduction rate

LDL-C goal attainment rates in all high-risk patients (known plus newly defined), defined according to target LDL-C level and stratified by disease, are shown in Fig. 4a. LDL-C goal attainment rates in stroke patients (*n* = 56,727) for < 70 mg/dL were 11.7%; and in ACS patients (*n* = 7788) were 26.3%. In CHD patients (*n* = 95,302), LDL-C attainment rates for < 70 mg/dL were 12.7%; and, in PAD patients (*n* = 145,574), were 9.2% (Fig. 4a).

Attainment rates for DM patients with a high-risk for CVD (*n* = 105,800) were higher for achieving < 70 mg/dL goals than for DM patients without high risk (*n* = 99,666) (12.2% vs. 8.3%, respectively). Attainment rates for ≥70 to < 100 mg/dL (27.1% vs. 24.3%) were comparable. Similarly, a higher proportion of patients with AAD with high-risk (*n* = 5415) achieved < 70 mg/dL goals than patients without high risk (*n* = 1409) (20.9% vs. 11.8%). Respective attainment rates for ≥70 to < 100 mg/dL were 32.4% versus 28.7% (Fig. 4a).

In newly defined high-risk patients, LDL-C goal attainment was defined as an LDL-C reduction > 50%, according to ACC/AHA guidelines [3] (Fig. 4b). These goals were achieved in 34.5% of 6981 stroke patients, 57.8% of 1933 ACS patients, 35.5% of CHD patients (*n* = 12,021), 25.4% of PAD patients (*n* = 15,136), 31.4% of DM patients (*n* = 16,801), and by 35.1% of AAD patients (*n* = 2836) (Fig. 4b).

**Fig. 5:**
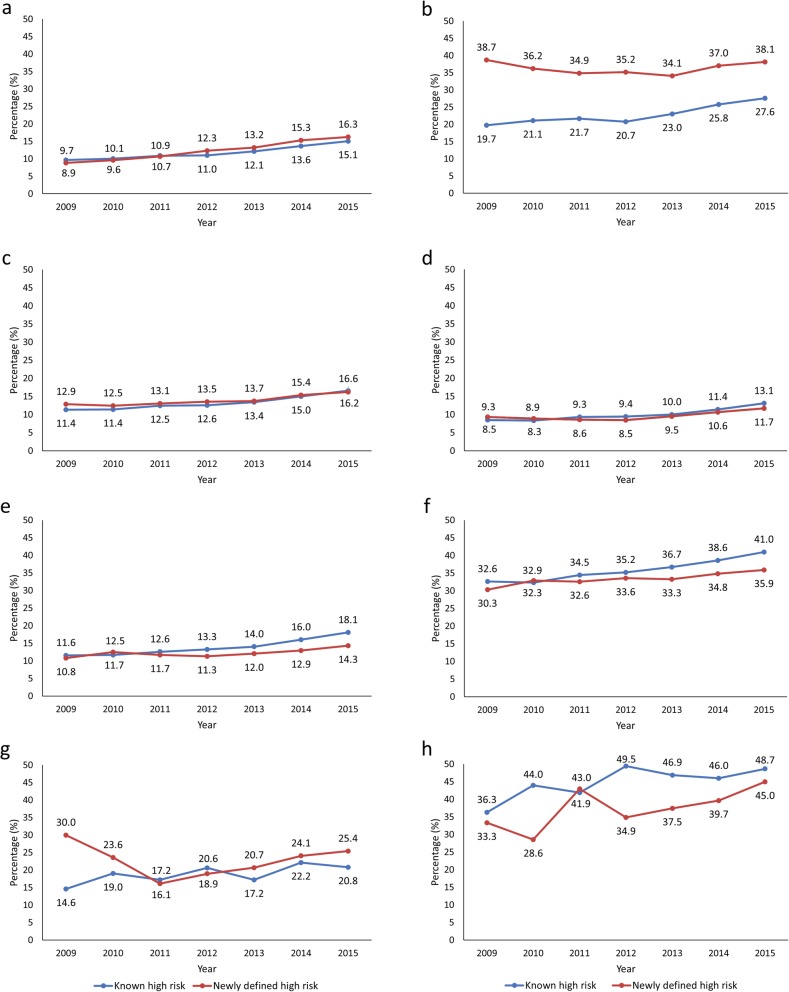
Time trends of goal attainment in known and newly defined high-risk patients with **a.** stroke; **b.** ACS; **c.** CHD; **d.** PAD; **e.** DM with high risk of cardiovascular disease (CVD); **f.** DM without high risk of CVD; **g.** AAD with high risk of CVD; **h.** AAD without high risk of CVD. *P* for trend of known high-risk and newly defined high-risk, respectively. **a.** < .0001 and < .0001; **b.** <.0001 and 0.1081; **c.** <.0001 and < .0001; **d.** <.0001 and < .0001; **e.** <.0001 and < .0001; **f.** <.0001 and < .0001; **g.** 0.0027 and < .0001; **h.** 0.0226 and 0.0138

Time trends of LDL-C goal attainment in known and newly defined high-risk patients in each disease group are shown in Fig. 5. In stroke patients, there were similar upward trends from 2009 to 2015 in both known (from 9.7 to 15.1%) and newly defined (from 8.9 to 16.3%) high-risk patients. In contrast, in ACS patients, the proportion of known high-risk patients achieving LDL-C goals increased steadily from 2009 to 2015 (from 19.7 to 27.6%), whereas the proportion of newly defined high-risk patients remained reasonably constant (38.7% in 2009 and 38.1% in 2015).

In CHD and PAD patients, the time-trend curves for known and newly defined high-risk patients were virtually superimposable. In CHD patients, LDL-C goal attainment steadily increased from 2009 to 2015: from 11.4 to 16.6% in known, and 12.9 to 16.2% in newly defined high-risk patients. In PAD patients, goal attainment rates from 2009 to 2015 were 8.5 to 13.1% in known, and 9.3 to 11.7% in newly defined high-risk patients (Fig. 5).

Time trends in DM patients with or without high-risk showed that DM patients without high-risk consistently had higher LDL-C goal attainment rates than DM patients with high-risk, irrespective of the known or recent diagnosis of high-risk. Attainment rates in 2015 for DM patients without high-risk were 41.0 and 35.9%, respectively, for known and newly defined high-risk groups, compared with rates of 18.1 and 14.3% for newly defined high-risk groups in DM patients with high-risk (Fig. 5).

Attainment rates of atherosclerosis patients with high-risk were generally similar in newly defined high-risk patients (16.1% in 2011, 25.4% in 2015) compared with known high-risk patients (14.6% in 2011, 20.8% in 2015), although the attainment rate tended to be higher in newly defined high-risk than in known high-risk in 2009. However, in atherosclerosis without high-risk, attainment rates were generally higher in known high-risk patients (36.3% in 2009, 48.7% in 2015) than in newly defined high-risk patients (33.3% in 2009, 45.0% in 2015) (Fig. 5).

## Discussion

This retrospective study using the Korean NHIS-HEALS large number database addressed that dyslipidemia management in patients with high-risk for CVD needs to be improved. Although ACS patients who were newly defined high-risk group was the most controlled among the groups (34.4%), the control of LDL-C levels is still not good enough considering consequence CVD risks and its disease burden. Based on the key findings from this study, LDL-cholesterol level reduction treatment strategies and “treat to-target” groups need to be clarified.

Statin use was highest in patients with ACS or AAD. Overall, 49.4 and 78.0% of ACS patients with known and newly defined high-risk received statins, respectively, whereas respective figures for patients with AAD were 44.2 and 61.3%. A recent US study found that, although around 90% of high-risk patients started treatment with statin monotherapy, treatment initiation with high-intensity statins was ≤10% [27]. In present study, most patients received moderate-intensity statin therapy; in general, < 10% of patients received high-intensity statin therapy. The exception was ACS patients with newly defined high-risk, of whom 24.9% received high-intensity statin therapy.

Guidelines for lipid management in ACS patients vary, and target values, have changed in recent years. 2013 ACC/AHA and 2016 ACC Expert Consensus Guidelines recommend high-intensity statin therapy, which lowers LDL-C levels on average by approximately ≥50%, and moderate-intensity statin therapy, which lowers LDL-C on average by approximately 30 to < 50% for patients aged > 75 years or who are not candidates for high-intensity statin therapy [3, 28]. These guidelines are also applicable to patients with stroke or other clinical ASCVD events [3, 28]. Current 2018 Korean guidelines recommend LDL-C treatment goals dependent on risk assessment: very high-risk < 70 mg/dL, high-risk < 100 mg/dL, moderate-risk < 130 mg/dL, and low-risk < 160 mg/dL [23]. Using 2013 ACC/AHA guideline target values [3], goal attainment rates were relatively high compared to those using the 2018 Korean guidelines, even in the newly defined high-risk patient group (data not shown). For example, in ACS, LDL-C goal attainment rate was 26.3% by target LDL-C level and 57.8% by reduction rate. One possible explanation is that doctors treating high-risk patients consider that LDL-C 100 mg/dL is a sufficiently low attainment level for dyslipidemia treatment, although data derived from KNHANES (2014–2016) show that 17.6% of adult Koreans (aged ≥30 years) had hyper-LDL cholesterolemia (LDL-C ≥ 160 mg/dL) [8]. On the other hand, the higher attainment rate by reduction rate than by target LDL-C level, even though most high-risk patients received moderate intensity statin, may be related to higher statin efficacy in Asians compared to Caucasians [29–32]. Further studies are warranted for more appropriate secondary prevention in high-risk patients.

Time-trend (2009–2015) for LDL-C goal attainment were similar for comparisons of known and newly defined high-risk patients with stroke, CHD, PAD, AAD with additional high-risk disease or DM with/without additional high-risk disease. In ACS, newly defined high-risk patients had consistently higher attainment rates from 2009 to 2015 compared to known high-risk patients. Although reasons for differences were not assessed in this study, they possibly reflect patient medication adherence issues [33, 34], and/or suboptimal performance and poor perception of physicians regarding attainment rates [35], resulting in patients receiving inadequate dosages or titration of lipid-lowering medication [36, 37]. On the other hand, even though the achievement rate was relatively high in newly defined ACS patients, the gap had been narrowing due to no improvement in newly defined ACS patient. It also reflects suboptimal perception of physicians [35] in newly diagnosed cases, but, further consideration is needed for causes of no improvement of goal attainment in newly defined ACS patients, in contrast to the increase in known ACS patients.

There have been clinical practice changes due to changes in the guidelines such as ACC/AHA [3] and ESC/EAS [15] since the NCEP-ATP III guideline [2]. It was expected that the use of high-intensity statins and the achievement of LDL-C targets in the groups of the high-risk and very high-risk increased. Although the time trends of LDL-C goal attainment had been generally increasing in all groups except newly defined ACS patients, most patients did not achieve LDL-C targets. Attainment rates were < 50% for patients in each disease category, including the best LDL-C target attainment shown in ACS patients. Even in a previous Korean study of diabetic patients treated by specialists, the LDL-C goal attainment in patients receiving lipid-lowering therapy was low at 47.4% in 2010 [35]. Comparable results (for 2010) in this study showed LDL-C goal attainment rates of approximately 12 and 32%, in DM patients with or without additional high-risk disease, respectively. Low rates of LDL-C goal attainment have also been described consistently in other countries, including 58% of recent ACS patients in the Netherlands [38], 28.8% of ACS survivors in Hong Kong and Taiwan [39], 30% of German atherosclerotic CVD patients [40], 41% of patients with DM at very-high cardiovascular risk receiving statins in France [41] and 38% of DM patients with ischemic heart disease in a tertiary hospital in China [42]. In contrast, a higher attainment rate (68%) for Japan Atherosclerosis Society guideline-recommended LDL-C targets [43] was reported in high-risk patients for CVD in Japan [44].

This study has several strengths. The current study is derived from a nationally-representative cohort of Korean individuals (NHIS-HEALS database, *n* = 514,866) with a relatively low attrition rate [24], reflecting real-world clinical circumstances. Because information on drug use and bio-clinical laboratory results were included in databases, the risk of recall bias was eliminated. Considering the lack of data in Asian populations for estimating LDL-C goal achievement, results of this study provide information on possible LDL-C reduction rates. In addition, the estimation of goal attainment rate by LDL-C level (< 70 mg/dL, 70–100 mg/dL for target goal) and by percent of LDL-C reduction, together in a large national cohort, is valuable.

However, this study also has some limitations. The result needed to be interpreted with consideration of followings; Subjects were selected based on the availability of LDL-C measurements, which may limit the generalizability of results to general population. Additionally, the age of participant limited between 40 and 70 years and it may not be applicable to goal attainment of young adult participants, or oldest-old participants. In addition, due to the nature of the NHIS-HEALS database, disease diagnosis variables may reflect healthcare usage which is sensitive to the fee-for-service payment and reimbursement system in Korea, rather than being an accurate reflection of a patient’s specific medical condition.

## Conclusions

LDL-C goal attainment rates in Korean patients with CVD or with a high-risk for CVD are still poor, with < 50% of patients achieving LDL-C targets. Proactive action is needed to improve dyslipidemia management in high-risk patients with CVD, including those with stroke or ACS.

## Data Availability

The National Health Insurance Service-National Health Screening Cohort (NHIS-HEALS) was third party data owned by the National Health Insurance Corporation (NHIC). Interested researchers can contact NHIC to access the data in the following ways: Tel: 82–33–736-2469 (Big data operation room, NHIC), Web: https://nhiss.nhis.or.kr/bd/ab/bdaba006cv.do.

## Abbreviations

AAD: Atherosclerotic artery disease
ACC: American College of Cardiology
ACS: Acute coronary syndrome
AHA: American Heart Association
ASCVD: Atherosclerotic cardiovascular disease
BMI: Body mass index
CHD: Coronary heart disease
CVD: Cardiovascular disease
DM: Diabetes mellitus
EAS: European Atherosclerosis Society
ESC: European Society of Cardiology
HDL-C: High-density lipoprotein cholesterol
ICD: International Classification of Disease
IRB: Institutional review board
KNHANES: Korean National Health and Nutrition Examination Survey
LDL-C: Low-density lipoprotein cholesterol
NCEP-ATP: National Cholesterol Education Program-Adult Treatment Panel
NHIS-HEALS: National Health Insurance Service-National Health Screening Cohort
PAD: Peripheral artery disease
SD: Standard deviation
